# Astrocyte Hypertrophy and Microglia Activation in the Rat Auditory Midbrain Is Induced by Electrical Intracochlear Stimulation

**DOI:** 10.3389/fncel.2018.00043

**Published:** 2018-02-22

**Authors:** Nicole Rosskothen-Kuhl, Heika Hildebrandt, Ralf Birkenhäger, Robert-Benjamin Illing

**Affiliations:** ^1^Neurobiological Research Laboratory, Section for Clinical and Experimental Otology, University Medical Center Freiburg, Freiburg, Germany; ^2^Molecular Biological Laboratory, Section for Clinical and Experimental Otology, University Medical Center Freiburg, Freiburg, Germany

**Keywords:** neuroplasticity, glioplasticity, inferior colliculus, hearing experience, cochlear implant

## Abstract

Neuron–glia interactions contribute to tissue homeostasis and functional plasticity in the mammalian brain, but it remains unclear how this is achieved. The potential of central auditory brain tissue for stimulation-dependent cellular remodeling was studied in hearing-experienced and neonatally deafened rats. At adulthood, both groups received an intracochlear electrode into the left cochlea and were continuously stimulated for 1 or 7 days after waking up from anesthesia. Normal hearing and deafness were assessed by auditory brainstem responses (ABRs). The effectiveness of stimulation was verified by electrically evoked ABRs as well as immunocytochemistry and *in situ* hybridization for the immediate early gene product Fos on sections through the auditory midbrain containing the inferior colliculus (IC). Whereas hearing-experienced animals showed a tonotopically restricted Fos response in the IC contralateral to electrical intracochlear stimulation, Fos-positive neurons were found almost throughout the contralateral IC in deaf animals. In deaf rats, the Fos response was accompanied by a massive increase of GFAP indicating astrocytic hypertrophy, and a local activation of microglial cells identified by IBA1. These glia responses led to a noticeable increase of neuron–glia approximations. Moreover, staining for the GABA synthetizing enzymes GAD65 and GAD67 rose significantly in neuronal cell bodies and presynaptic boutons in the contralateral IC of deaf rats. Activation of neurons and glial cells and tissue re-composition were in no case accompanied by cell death as would have been apparent by a Tunel reaction. These findings suggest that growth and activity of glial cells is crucial for the local adjustment of neuronal inhibition to neuronal excitation.

## Introduction

In the mammalian brain, any pattern of signaling activity is molding the substrate that carries it. The substrate is an intricate meshwork of neurons, glial cells, and extracellular structures and substances, all of which come in a considerable variety of types and states. The study of the phenomenon of brain plasticity is of importance throughout the neurosciences, but whereas there is a huge body of literature about the plasticity of neurons and their synapses, much less is yet understood about the other actors (Reemst et al., 2016).

One of the first indicators of reactive cellular modification due to neural network activity is the expression of the FBJ osteosarcoma oncogene *fos*, also known as *c-fos*, in all parts of the mammalian central nervous system. Fos is often just used as a marker for spiking intensity indicating neuronal activity (Cohen and Greenberg, 2008; Cruz et al., 2013), but its deeper involvement is gradually acknowledged. As a monomer of the heterodimeric Fos:Jun activator protein-1 (AP-1) transcription factor, Fos may trigger the expression of specific genes that contribute to functional and structural alterations of the affected neurons and their surroundings. If no Fos is available, AP-1 rather comes in the pATF2:Jun variant, which activates a different set of genes (Rauch et al., 2016).

In the central auditory system, Fos may be induced by acoustical or electrical intracochlear stimulation (EIS). Strong and long lasting stimulation is accompanied by a strengthening of inhibitory interactions within these networks (Argence et al., 2008). Conversely, if sensory excitation is experimentally prohibited by bilateral deafening, inhibitory interactions are weakened (Bledsoe et al., 1995; Abbott et al., 1999; Vale and Sanes, 2002; Vale et al., 2003), so as if the level of excitation re-balances neuronal inhibition.

The subtle, synapse-wise adjustment of neuronal excitation and inhibition is known to be essential for coding and processing of sensory signals adequate for adapted behavior (Tao and Poo, 2005; Li and Pollak, 2013). It is as yet not understood how this adjustment is achieved by cellular and molecular signaling. In order to inquire deeper into the connectedness of neural and glial plasticity, this study is based on experiments of the central auditory system of adult rats with different hearing experience. Here, one purpose was to use the expression of Fos protein and *fos* mRNA for probing the state of the central inferior colliculus (CIC). The CIC has been termed hub of the auditory brainstem (Casseday et al., 2002) since virtually all processing lines originating in various lower and higher auditory nuclei converge upon its central nucleus (Lee and Sherman, 2011). Based on this arrangement, new and complex response properties emerge in collicular cells on which all subsequent processing of auditory signals depends.

Astrocytes play important roles in neuronal function (Reemst et al., 2016) and synaptic plasticity (Kang et al., 1998), both extending into cognitive functions (Ben Achour and Pascual, 2012). Astrocytes, like neurons, exhibit several forms of plasticity, including morphological changes (Genoud et al., 2006; Oliet and Bonfardin, 2010) and functional modifications by neuronal-induced currents. Astrocytes are affected by neuronal activity through their membrane channels, receptors, and transporters. They release glutamate, ATP, D-serine, and lactate to play signals back to modulate activity in surrounding cells (Nedergaard and Verkhratsky, 2012; Stehberg et al., 2012).

Microglia, frequently referred to as the immune cells of the brain, may exert neurotoxic or neuroprotective effects, phagocyte cellular debris, modulate astroglial physiology, and participate in synaptic plasticity in a stimulus-dependent manner (Ransohoff and Perry, 2009; Graeber and Streit, 2010). In the adult brain, resting microglia were shown to influence basal neurotransmission (Hayashi et al., 2006) and appear to be essential when neuronal reorganization is required (Rappert et al., 2004).

A major question is how neuronal firing activity is linked to a reactive strengthening of GABAergic transmission in local cellular networks. We here suggest that both astrocytes and microglia are essential components forming this link. To investigate this issue, we chose to use the cochlear implant as an experimental tool for full control of stimulation parameters, probing with it state and responsiveness of the adult central auditory system depending on the history of its hearing experience. This report focuses on the CIC as it is the auditory brainstem site of major neuro–glia responses to our experimental interventions.

## Materials and Methods

### Animals

This study is based on the analysis of 43 brains from female Wistar rats aged 8–12 weeks. Care and use of the rats as reported here conformed to the Declaration of Helsinki (1964, 1989) and were approved by the appropriate agency (Regierungspräsidium Freiburg, permission number G-10/83).

### Anesthesia

For recording the auditory brainstem response (ABR), ear bone removal or cochlear opening, and electrode insertion, rats were anesthetized by intraperitoneal (i.p.) injection of a mixture of ketamine (Medistar Arzneimittelvertrieb GmbH, 80 mg/kg body weight) and xylazine (Rompun; Bayer Vital GmbH, 12 mg/kg body weight). For pain reduction, rats received a subcutaneous injection of Carprofen (Carprieve; Norbrook Laboratories Ltd., 4 mg/kg body weight). Preceding transcardial perfusion, rats were given a lethal dose of sodium-thiopental (Trapanal; Nycomed, 250 mg/kg body weight), leading to respiratory arrest.

### Experimental Groups

Rats were divided in total into eight experimental groups. Hearing-experienced rats were divided into four groups: zero controls, i.e., animals experiencing no experimental manipulation at all (*n* = 4), implantation controls, i.e., animals that received complete surgery including electrode insertion but were not stimulated [*n* = 3 for 1 day (d), *n* = 5 for 7 d], animals stimulated for 1 d (*n* = 5), or for 7 d (*n* = 4). Correspondingly, neonatally deafened rats were also divided into four groups: zero controls, i.e., animals that experienced no experimental manipulation except neonatal deafening as described in the next paragraph (*n* = 3), implantation controls (*n* = 5 for 1 d, *n* = 3 for 7 d), animals stimulated for 1 d (*n* = 7), and animals stimulated for 7 d (*n* = 4).

### Neonatal Deafening

Rats were neonatally deafened by daily i.p. injections of kanamycin sulfate from *Streptomyces kanamyceticus* (Sigma, Cat. No. K0254, 400 mg/kg body weight) from postnatal day 10–20, inclusively, causing pronounced damage on almost every outer hair cell throughout the turns of the cochlear in response to the exposure of the ototoxic antibiotic (Osako et al., 1979; Matsuda et al., 1999). Hearing loss by over 80 dB SPL was quickly indicated by the loss of Preyer’s reflex (Jero et al., 2001).

### Auditory Brainstem Response (ABR)

**FIGURE 1:**
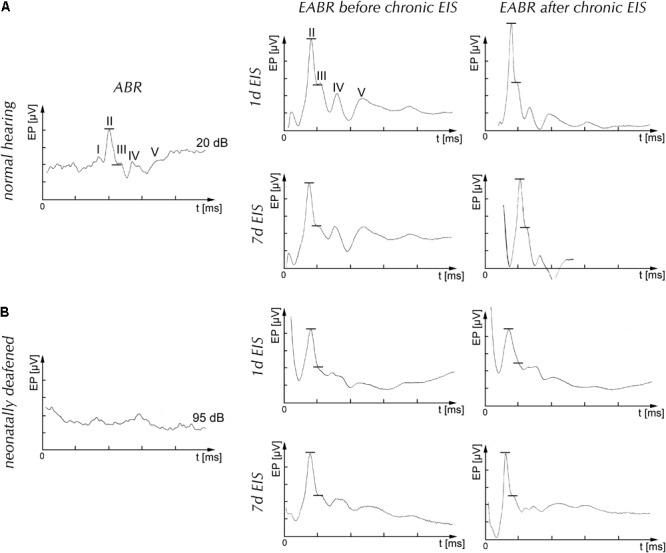
Auditory brainstem responses after acoustical (ABR) or electrical (EABR) stimulation of the cochlea of normal hearing (nh) **(A)** and neonatally deafened **(B)** adult rats. Left column of **(A)** and **(B)**: acoustical stimulation evoked a typical brainstem response in nh rats with five differentiated peaks I–V (upper graph), but failed to generate a response even at 95 dB above nh threshold in deafened rats (lower graph). Middle column of **(A)** and **(B)** shows representative measurements. Before the onset of chronic EIS, the EABRs were recorded to verify effective stimulation. Immediately preceding termination of experiments, EABRs were again recorded to verify intactness of set-up and effectiveness of EIS. R column of **(A)** and **(B)** shows representative measurements. Roman numbers list positive peaks of ABR and EABR; line segments indicate site of amplitude measurement; d, day/days; EIS, electrical intracochlear stimulation; EP, evoked potential.

For ABR recording, insulated and conically tipped brass pipes were introduced into both outer ear canals, followed by subcutaneous placement of steel needle electrodes at the vertex and bilaterally at mastoids. Ears were stimulated and recorded separately by presenting a 20 Hz sequence of click stimuli to one ear, while masking the contralateral ear with noise at the same sound pressure level. Stimulation intensity was stepwise increased to elicit an ABR graph visualized by an averager (Multiliner E, Evolution 1.70 c; Toennies and Jäger GmbH). Hearing threshold was identified as the first unequivocal response emerged. ABR mean amplitudes were determined by averaging 300 sweeps in a frequency band of 0.1–3 kHz. For deafened rats, acoustic stimulation went up to 95 dB above normal hearing (nh) threshold (**Figures 1A,B**). When acoustic stimulation failed to produce an ABR, stimulation was discontinued and the threshold was noted to be above 95 dB.

### Proof of Deafness

Confirming results of Matsuda et al. (1999) and Osako et al. (1979), we determined ABR thresholds bilaterally increasing by around 92 dB for rats neonatally deafened by systemic kanamycin treatment which is a result of pronounced outer hair cell damage in every turn of the cochlea (Osako et al., 1979). Additionally, all rats consistently failed to show a motor response to a handclap. The absence of this so-called Preyer’s reflex was taken by Jero et al. (2001) in mice as indicating a rise of ABR threshold beyond 81 dB SPL.

### Electrical Intracochlear Stimulation (EIS)

Details of surgical approach, stimulation cage, and stimulation equipment are described in detail in previous studies (Rosskothen-Kuhl and Illing, 2010, 2012, 2014). In short, for the unilateral EIS, two rings of an electrode array with eight contacts (ST08.45, Cochlear Ltd.) were inserted through a cochleostomy in a medio-dorsal direction into the middle turn of the left cochlea and connected to the external stimulator by way of a swivel (Rosskothen-Kuhl and Illing, 2014). Still under anesthesia, the electrodes were activated to generate an electrically evoked auditory brainstem response (EABR).

Biphasic stimuli of 50 Hz with a phase width of 50 μs and an interphase gap of 25 μs were used in bipolar electrode stimulation. EABRs were recorded to verify correct placement of the electrode array and the effectiveness of stimulation. They were visualized by the averager, calculating mean amplitudes over 500 sweeps (**Figure 1**). EIS current levels were set to produce EABR amplitudes of 9 μV ± 10% that corresponded to acoustic stimulation of about 90 dB above hearing threshold of nh rats, causing specific tonotopic activation of central auditory neurons (Rosskothen et al., 2008; Illing and Rosskothen-Kuhl, 2012; Rosskothen-Kuhl and Illing, 2012). The term tonotopy refers to the topological neighborhood of neurons that preferentially respond to tones of similar frequency, spreading out frequency maps in essentially all parts of the central auditory system of nh mammals.

After rats recovered from anesthesia, electrodes were activated for causing local activation of the spiral ganglion. EIS was conducted for durations of 1 or 7 d on unanesthetized rats without interruption. Immediately preceding termination of the experiments, rats were re-anesthetized and the EABR was recorded again to verify intact components and efficient stimulation for the time period envisioned (**Figure 1**, right column). In control experiments, the electrode carrier was inserted into the cochlea for 1 or for 7 d without being activated.

### Immunohistochemistry

Following completion of postoperative survival time or stimulation period, rats received a lethal dose of sodium-thiopental and were transcardially perfused with a fixative containing 4% PFA in 0.1 M phosphate buffer (PB) at pH 7.4. After the brain was removed from the skull and soaked in 20% sucrose in PB overnight, parts containing the CIC were cryo-cut into 30 μm thick frontal sections. Following incubation with 0.045% H_2_O_2_ and 1% milk powder, each in 0.02 M PBS at pH 7.4 for 30 min, sections were exposed to a primary antibody raised in goat against Fos protein, in mouse against GFAP, in rabbit against S100, in rabbit against IBA1, in mouse against GAD65, in mouse against GAD67, in rabbit against GABA, in mouse against HuC/HuD, or in guinea pig against vGluT1 (**Table 1**). After incubation for 48 h at 4°C, a species matching biotinylated secondary antibody was added. Visualization of primary antibody binding sites was based on the avidin–biotin technique (Vector Laboratories, Cat. No. PK-6100), followed by incubation in DAB (Sigma, Cat. No. 32750) in 0.3% ammonium nickel(II) sulfate hexahydrate (Sigma, Cat. No. A1827) in Tris buffer containing 7.5% sucrose. Controls omitting primary antibodies were run to verify their specificity and lack of unspecific binding by the secondary antibody (not shown).

**Table 1:** Primary and secondary antibodies used.

Antigen	Species of origin	Company/Order No.	Dilution (DAB/FL)	Address of company
**Primary antibodies**				
Fos	gt	Santa Cruz/sc-52	1:2000/1:100	Santa Cruz Biotechnology, Inc., Dallas, TX, United States
GFAP	mo	Sigma/G3893	1:5000/1:5000	Sigma–Aldrich Chemie GmbH, Munich, Germany
GAD65	mo	Millipore/MAB351	1:2000/1:500	Merck Chemicals GmbH, Darmstadt, Germany
GAD67	mo	Millipore/MAB5406	1:5000/1:500	Merck Chemicals GmbH, Darmstadt, Germany
vGluT1	gp	Millipore/AB5905	1:5000/1:1000	Merck Chemicals GmbH, Darmstadt, Germany
IBA1	rb	Wako/019-19741	1:1000/1:500	Wako Pure Chemical Industries, Ltd., Osaka, Japan
HuC/HuD	mo	Invitrogen/A-21271	- /1:100	Thermo Fisher Scientific Inc., Darmstadt, Germany
GABA	rb	Sigma/A2052	- /1:2000	Sigma–Aldrich Chemie GmbH, Munich, Germany
S100	rb	Dako/Z0311	- /1:400	DAKO, Agilent, Santa Clara, CA, United States
**Secondary antibodies for DAB staining**				
gt IgG	rb/HRP	Vector/BA-5000	1:200	Vector Laboratories, Burlingame, CA, United States
mo IgG	ho/HRP	Vector/BA-2001	1:200	Vector Laboratories, Burlingame, CA, United States
rb IgG	gt/HRP	Vector/BA-1000	1:200	Vector Laboratories, Burlingame, CA, United States
gp IgG	gp/HRP	Vector/BA-5003	1:200	Vector Laboratories, Burlingame, CA, United States
**Secondary antibodies for immunofluorescence staining**				
gt IgG	dk/Cy3	Millipore/AP180C	1:200	Merck Chemicals GmbH, Darmstadt, Germany
rb IgG	dk/Alexa488	Molecular Probes/A-21206	1:200	Thermo Fisher Scientific Inc., Darmstadt, Germany
mo IgG	dk/Alexa488	Molecular Probes/A-21202	1:200	Thermo Fisher Scientific Inc., Darmstadt, Germany
rb IgG	gt/Biotin	Vector/BA-1000	1:200	Vector Laboratories, Burlingame, CA, United States
Streptavidin	Amca	Vector/SA-5008	1:33	Vector Laboratories, Burlingame, CA, United States

Double or triple immunofluorescence was achieved on free-floating sections pre-treated with 0.1% Triton X-100 in PBS and serially incubated with two or three primary antibodies for 20 h each at 4°C. Subsequently, incubations of matching secondary antibodies for 1 h were performed serially, preceded by pre-incubation with 5% donkey serum each lasting for 30 min (**Table 1**). Media for all antibodies and sera contained 0.05% Triton X-100. Stained sections were mounted on gelatin-coated glass slides, air-dried overnight, and cover slipped with M-Glas (Merck, Cat. No. 1039730001).

### *In Situ* Probes

For *fos* fragment synthesis, *fos* mRNA was isolated from adult rat cochlear nucleus after 2 h EIS. Subsequent cDNA synthesis was performed using standard techniques (Qiagen, Omniscript RT, Cat. No. 205111). Based on the cDNA, a 313 base pairs (bp) long DNA fragment of the *fos* gene (NCBI Sequence Read Archive, NM_022197, GI: 148298807) was amplified with the following primers: F1-FBJ/*fos* (5′-AGCTCCCACCAGTGTCTACC-3′) and R1-JB/*fos* (5′-CCACGGAGGAGACCAGAGTG-3′).

For riboprobe construction, the *fos* fragment was subcloned into pCR4-TOPO (Invitrogen, Cat. No. K459501) to construct subclone pCR4 *fos*. From this construct a linearized *fos* fragment flanked by the T3 and T7 promoters was amplified. For both fragments, digoxigenin (DIG)-labeled antisense riboprobes were generated from the 313 bp rat *fos* cDNA after transcription with T3 RNA polymerase (Roche, Cat. No. 11031163001). Sense riboprobes were made after transcription with T7 RNA polymerase (Roche, Cat. No. 10881767001). These sense probes served as control to verify that the complementary transcript failed to generate staining.

### *In Situ* Hybridization

*In situ* hybridization was based on a protocol described in detail in Rosskothen-Kuhl and Illing (2014). In short, 30 μm thick cryo-cut frontal brain sections were collected in 2× SSC buffer (Gibco, Cat. No. 15557-044). Before pre-hybridization, they were pre-treated in a 1:1 dilution of 2× SSC and a hybridization buffer consisting of 50% formamide (Roth, Cat. No. P040.1), 4× SSC, 5% dextransulfate (Sigma, Cat. No. D8906), 1× Denhardt’s solution (Invitrogen, Cat. No. 750018), 250 μg/ml heat-denatured fish sperm DNA (Roche, Cat. No. 11467140001), and 100 μg/ml tRNA from *Escherichia coli* MRE 600 (Roche, Cat. No. 10109541001) for 15 min. Pre-hybridization in hybridization buffer at 55°C lasted for 60 min. Hybridization was done overnight at 55°C in the same solution with the addition of 100 ng/ml DIG-labeled *fos* antisense or sense RNA, respectively. For immunological detection of DIG-labeled hybrids, brain sections were treated twice in TBS (100 mM Tris/HCl, pH 7.5; 150 mM NaCl) for 10 min each, blocked in 1% blocking reagent (Roche, Cat. No. 11096176001) and incubated overnight at 4°C with the anti-DIG Fab fragment from sheep tagged with alkaline phosphatase (Roche, Cat. No. 11093274910 1:1500). NBT (Roche, Cat. No. 11383213001, 0.34 mg/ml) and BCIP (Roche, Cat. No. 11383221001, 0.17 mg/ml) were added to a buffer containing 100 mM Tris/HCl, pH 9.5; 100 mM NaCl; and 50 mM MgCl_2_. Development of the color reaction was done for 9 h. Color reaction was stopped by transferring sections into distilled water. Sections were mounted on glass slides, dehydrated, cleared in xylene, and coverslipped with DPX (Sigma, Cat. No. 06522).

### Tunel Staining

Nuclear DNA fragmentation in apoptotic cells was detected on cryo-cut brain sections by using a TACS-XL DAB *in situ* apoptosis detection kit (Trevigen, Cat. No. 4828-30-DK). BrdU incorporation by terminal deoxynucleotidyl transferase at the site of DNA fragmentation was detected by a highly specific and sensitive biotinylated anti-BrdU antibody and visualized by a streptavidin–horseradish peroxidase conjugate. These brain sections were counterstained with methyl green.

### Microscopy

Micrographs of DAB-stained sections were taken with a digital camera (Zeiss, AxioCam, acquisition software Axiovision) connected to a light microscope (Zeiss, Axiophot) at eight-bit gray tone depth under strictly identical settings for each antibody. Sections stained for immunofluorescence were taken by the same equipment or, in cases indicated in the corresponding figure legend, by a confocal microscope (Leica, TCS SP2).

### Statistical Analysis

The phenomena presented here, in particular for the deaf rats, are massive and unequivocal, allowing us to judge them on an ordinal scale. Changes in the expression levels of GFAP, vGluT1, GAD65, and GAD67 were quantified on photographs of three to five sections per rat by measuring the mean gray tone intensity of DAB staining in regions of interest (ROIs) centered in the CIC. Care was taken on positioning these ROIs always bilaterally symmetrical for corresponding values (**Figures 8A,B**).

For counting stained nuclei or cell bodies, three to five sections per rat through the anterior–posterior center of the CIC were used. For counting Fos-positive nuclei in contralateral CIC, 5× grayscale photographs were processed by Adobe Photoshop CS (Adobe Systems Inc.) and imported to iTEM (Olympus) to detect automatically Fos-positive nuclei in the ROI (**Figure 3B**), using a threshold for gray tone values of 205. For counting GAD67 positive cell bodies, the ROI was centered in the CIC and photographed in mirror-symmetrical position on both sides of the midbrain. As mentioned above, counting of positive nuclei or cell bodies was done for the different experimental groups. In order to normalize for interindividual variance and marginal differences in staining conditions we present staining intensities and cell body counts as ratios of contralateral-to-ipsilateral, resulting in values above 1 if the CIC contralateral to surgery and stimulation stained more intensely or contained more items than the CIC ipsilateral to the stimulated or only implanted ear.

For quantifying IBA1 staining, the percentage of staining area based on grayscale photographs was taken from sections of the right (r)/contralateral (c) side of CICs through a ×40 objective with a digital camera (Zeiss, AxioCam, and AxioVision). Aided by graphics software (Adobe Photoshop CS), global variations in staining intensity of CIC sections from different rats were compensated by setting the median of the background to 230. Corrected photographs were then imported to iTEM and the detection threshold for gray tone values was adjusted to 145. The ROI corresponded to the total image size and to the position of ROIs used for evaluating other staining patterns. The fraction of IBA1 staining was determined in all experimental groups.

For statistical testing, ratios and SEM were depicted in histogram bars. Depending on the number of factors which are potential sources of variation, significant differences among groups were determined by applying one-way or rather two-way ANOVA with significance level set to *p* < 0.05, followed by Tukey’s or Sidak’s multiple comparisons *post hoc* test with significance level set to *p* < 0.05. Significance levels are indicated as ^∗∗∗^*p* < 0.001, ^∗∗^*p* < 0.01, or ^∗^*p* < 0.05.

## Results

### ABRs and EABRs of Hearing-Experienced vs. Deaf Rats

Normal hearing function and the effectiveness of the deafening method were verified by determining hearing threshold, electrically evoked auditory brainstem potentials and, when applicable, electrically evoked potentials before (**Figures 1A,B**, middle column) and after 1 or 7 d of EIS (**Figures 1A,B**, r column) for each animal. Only cases conforming to these criteria entered further analysis. All rats of the hearing group showed nh on both ears before electrode implantation (**Figure 1A**), whereas the hearing threshold of the deafened rats was bilaterally increased on average by 91.8 dB with a SEM of 1.4 (**Figure 1B**).

### Neuron–Glia Co-activation after EIS

**FIGURE 2:**
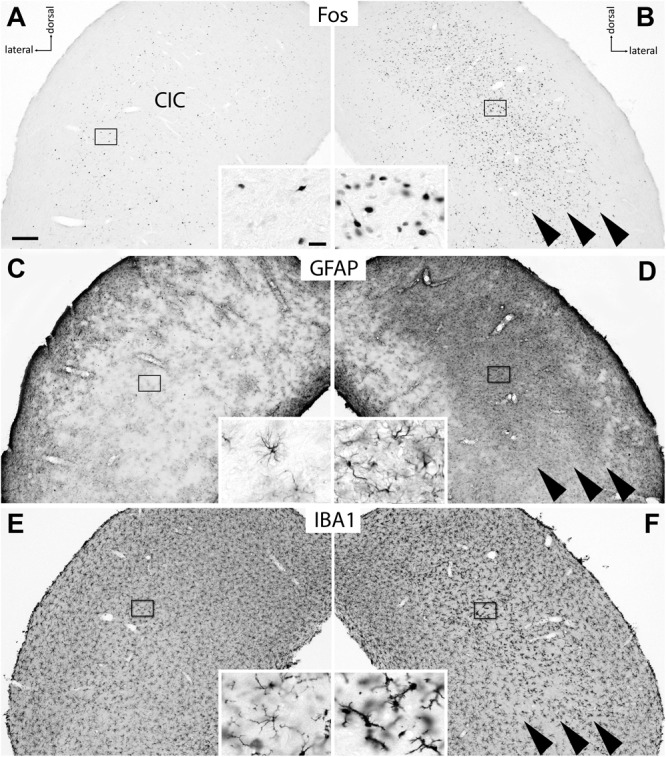
Effect of 1 d EIS on cells in the CIC of deafened rats. **(A,B)** Fos was expressed in the CIC contralateral to the simulated cochlea **(B)** in a population of neurons that extends beyond tonotopic boundaries known from nh animals (arrowheads). Ipsilaterally **(A)**, only few and scattered Fos-positive neurons were present. **(C,D)** In a nearby section stained for GFAP, a massive growth of astrocytes was noted taking place regionally specific in the contralateral CIC where Fos-positive neurons were abundant (**D**, arrowheads); no such changes occurred ipsilaterally **(C)**. **(E,F)** In another nearby section, microglia identified by IBA1 immunoreactivity showed a modified morphology indicating their activation in just the same region of the contralateral CIC (**F**, arrowheads); no such changes occurred ipsilaterally **(E)**. Frames show positions of insets. Scale bar for major panels: 200 μm; scale bar for insets: 20 μm.

After applying 1 d EIS to rats that grew up under nh or were neonatally deafened, we noticed massive cellular and molecular responses only in the CIC of hearing-inexperienced brains. **Figure 2** is provided as an overview of these major effects. The responses consisted of a widespread and massive instead of a focal expression of Fos in neurons (**Figure 2B**, arrowheads), the emergence of a densely aggregated astrocytes as revealed by GFAP staining (**Figure 2D**, arrowheads), and a marked change in the morphology of microglia indicated by IBA1 immunoreactivity indicating their transformation in an activated state (**Figure 2F**, arrowheads). The changes in these three types of cells occurred only in the CIC contralateral to the stimulated side, reflecting the crossing of the ascending auditory pathway to the opposite side before reaching the midbrain. No changes were observed ipsilateral to EIS (**Figures 2A,C,E**). The changes in the molecular profile of cells were spatially correlated and temporally staggered to each other, beginning with the expression of Fos by just over half an hour (Rosskothen-Kuhl and Illing, 2012), affecting most of the CIC with slightly stronger changes toward the dorsolateral edge of this nucleus for all three types of cells in the deafened rats. A marked difference existed in the glia responsiveness of CIC depending on the presence or absence of hearing experience.

### Absence of Molecular and Cellular Changes in Controls

**FIGURE 3:**
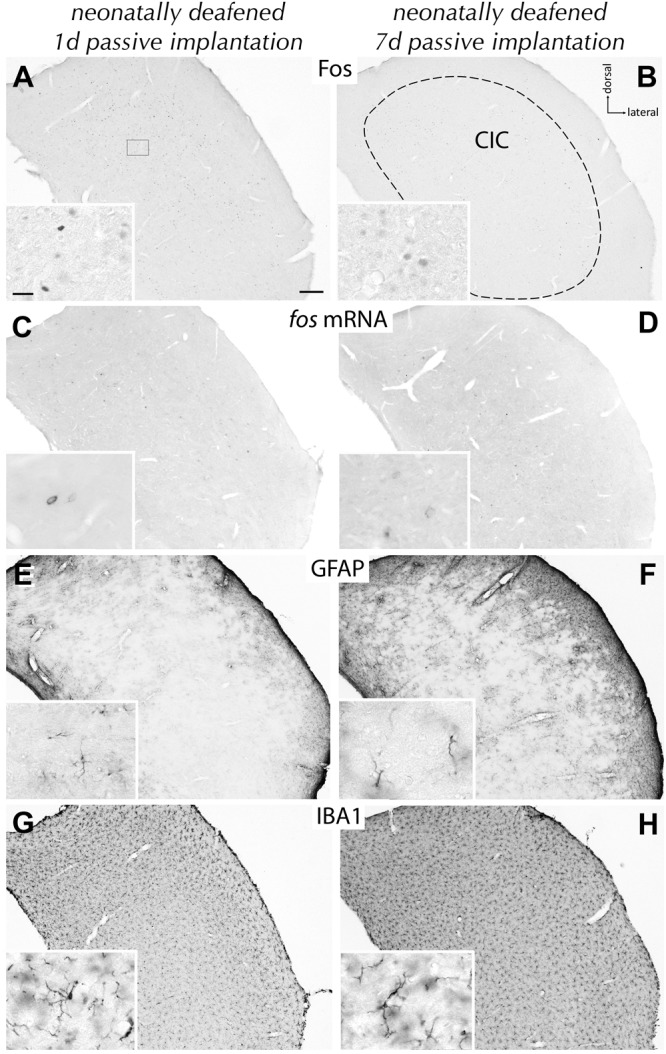
Absence of effects after passive intracochlear electrode implantation for 1 or 7 d. Staining of Fos protein **(A,B)**, *fos* mRNA **(C,D)**, GFAP **(E,F)**, or IBA1 **(G,H)** in the CIC contralateral to the intervention. After implantation of a passive electrode, intensity and pattern of staining for the markers used remained unchanged and indistinguishable from the ipsilateral (i) CIC or the CIC of untreated rats. Frame in **(A)** indicates approximate intracollicular position for all insets. Scale bar for major panels: 200 μm; scale bar for insets: 20 μm.

**FIGURE 4:**
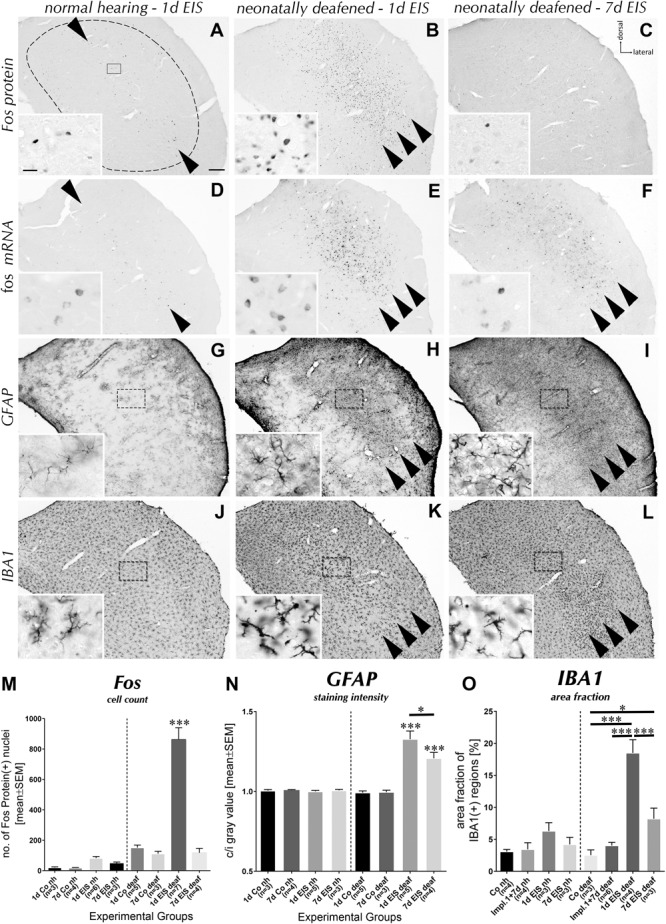
Effects of EIS in the contralateral CIC of hearing-experienced and deafened rats. **(A)** EIS induced Fos expression in a sharp tonotopically defined band across CIC (arrowhead) of nh rats. **(B)** Despite identical electrode position and stimulation parameters, Fos expression in deaf rats was extensive and spatially unrestricted within CIC after 1 d EIS (arrowheads). **(C)** After sustained EIS for 7 d of deaf rats, Fos expression was no longer where it has been before. **(D)** Like the protein, *fos* mRNA emerged in a tonotopic band of CIC after 1 d EIS of hearing-experienced rats (arrowhead). **(E)** Under otherwise identical stimulation conditions in deaf rats, *fos* mRNA was expressed by many neurons almost throughout CIC (arrowheads). **(F)** Unlike the protein, *fos* mRNA persisted even after 7 d EIS (arrowheads) of deafened rats. **(G)** GFAP staining remained apparently unchanged by EIS in hearing-experienced rats. **(H)** In deaf rats, a massive rise of GFAP staining intensity was seen in regions of high *fos* mRNA and protein expression (arrowheads). **(I)** GFAP staining was still elevated after 7 d of sustained EIS in deaf rats. **(J)** IBA1 staining remained apparently unchanged after EIS in hearing-experienced rats. **(K)** There was a striking change in microglia morphology after 1 d EIS of *(deaf rats in the region of high *fos* mRNA and protein expression (arrowheads). **(L)** By 7 d of EIS, microglia morphology was no longer as extreme as by 1 d EIS but still not back to normal. **(M)** Counting Fos protein positive cells in entire CIC cross-sections (dashed line in **A**), a significant rise was identified after 1 d EIS in hearing and deaf rats, which decreased again toward 7 d EIS. Group “1 d EIS deaf” differs significant (*p* < 0.001) against all other groups. Group “1 d EIS nh” differs significant against all other hearing groups with *p* < 0.001 or *p* = 0.027 for “1 d EIS” vs. “7 d EIS.” **(N)** The contralateral (c) to ipsilateral (i) ratio of GFAP staining intensity as determined in the frames indicated in G to I and their mirror-symmetrical counterparts in ipsilateral CIC was significantly shifted in favor of the contralateral side in deaf but not in hearing-experienced rats after 1 d EIS (*p* < 0.001 against all groups except “7 d EIS deaf” with *p* = 0.0333). By 7 d EIS, it has decreased again (^∗^ against “1 d EIS deaf”) but was still elevated against all other groups (*p* < 0.001). **(O)** Quantification of area fraction of IBA1 as determined in the frames indicated in **(J–L)** in contralateral CIC. A significant increase was identified after 1 d EIS of deaf rats, which decreased again toward 7 d EIS. Group “1 d EIS deaf” differs significant against all other groups (*p* = 0.016 for Co vs.7 d EIS deaf; *p* < 0.001 for all others). Co, Control with passive electrode implantation; nh, normal hearing; n, number of animals from which data were sampled. Scale bar for major panels: 200 μm; scale bar for insets: 10 μm.)*

Preparing for stimulation experiments, we needed to distinguish effects caused by surgery and/or by stimulation. After ossicle removal, opening of the cochlea via cochleostomy, and electrode insertion into the medial turn of the cochlea for 1 or 7 d resulting in acute deafness of the left ear of hearing rats, no changes in expression of Fos protein, *fos* mRNA, GRAP, or IBA1 were observed. Evaluating the brains of age-matched deaf rats that also received the full surgical procedures including electrode implantation but remaining unstimulated for 1 or 7 d, we identified no obvious effect on the expression of Fos protein (**Figures 3A,B**), *fos* mRNA (**Figures 3C,D**), GFAP (**Figures 3E,F**), or IBA1 (**Figures 3G,H**). Upon activation of the electrode, an entirely different scenario developed in the neonatally deafened group apparent by morphological and molecular changes of neurons, astrocytes, and microglia (**Figure 4**). These data provide the essential baseline to identify stimulation-dependent dynamics.

### Electrically Evoked Hearing Experience in Deaf Rats Induces Neuron–Glia Remodeling

Following 1 d (24 h) of chronic EIS of hearing-experienced rats (**Figure 4A**), the pattern of Fos protein staining was a fully plausible progression from what has already been documented for acute EIS (Rosskothen-Kuhl and Illing, 2010; Rauch et al., 2016). In sections through the CIC of these brains, there was a sharp line of cells containing the Fos protein in their nuclei (**Figure 4A**, black dots between arrowheads). This line was located in the 8–16 kHz area of the CIC (Ryan et al., 1988), corresponding tonotopically to the intracochlear position of the stimulating electrode implanted into the middle turn of the contralateral cochlea. Observations made on collicular sections from deaf rat brains revealed a very different outcome of EIS. Despite identical stimulation parameters and intracochlear stimulation position, Fos was expressed in a widespread population of cells extending almost throughout the contralateral CIC after 1 d of EIS, i.e., failing to show tonotopic order (**Figure 4B**, arrowheads; cp. Wu et al., 2003; Rosskothen-Kuhl and Illing, 2012). No comparable stimulation effect was identified in the ipsilateral CIC (**Figure 2A**). When EIS continued for another 6 d, expression of the Fos protein has declined (**Figure 4C**), reflecting both the limited half-live of the protein as well as the discontinuation of its production under monotone sustained activation.

Similar and spatially matching effects were observed when looking for *fos* mRNA (**Figures 4D–F**). Upon stimulation of hearing brains, it emerged in the contralateral colliculus in a narrow band that indicated tonotopic order (**Figure 4D**, arrowheads), but was induced in many more cells almost throughout CIC in deaf brains despite identical stimulation conditions (**Figure 4E**, arrowheads). The *fos* mRNA was observed for up to 7 d with decreasing intensity (**Figure 4F** and **Supplementary Figures S1, S3**). In no case was there a stimulation-dependent effect in the ipsilateral colliculus (indistinguishable from the staining in **Figures 3C,D**, not separately illustrated).

Adjacent or close sections of the CIC obtained from the same brains were stained for the astrocytic marker GFAP (**Figures 4G–I**). Staining of inferior collicular tissue from brains of hearing-experienced rats remained inconspicuous on both sides of the midline after either stimulation time (**Figure 4G**), displaying a pattern indistinguishable from control brains. By sharp contrast, staining intensified due to the growth of astrocytes that are now densely aggregated in the colliculus of deaf brains contralateral (**Figures 4H,I**, arrowheads) but not ipsilateral to EIS after either stimulation time. This astrocytic meshwork was confined to the region where cells containing Fos protein and *fos* mRNA were abundant and it persisted for days (**Figure 4**).

Staining the colliculus for the microglia marker IBA1 revealed comparable local modifications. Upon EIS for 1 d, nothing appeared to change in the CIC of hearing-experienced rats, in which a population of finely ramified microglial cells pervades the entire colliculus (**Figure 4J**). However, when deaf rats experienced 1 d of EIS, microglia of the contralateral CIC were seen to have altered their morphology to stout forms with a markedly reduced branching complexity (**Figure 4K**, inset), resulting in a reduced density of microglial arborization (**Figures 2F, 4K**, arrowheads). This occurred only in the region where the number of cells containing Fos protein rose dramatically. By 7 d of EIS, the microglia population has partially recovered, now showing cells that are still compact but appear to carry again finer processes (**Figure 4L**, arrowheads and inset). Furthermore, compared to deafened control animals (**Figures 3G,H**) and 1 d stimulated deaf rats (**Figure 4K**), the microglial network seems to be denser in 7 d stimulated deaf rats (**Figure 4L**). Neuron–glia responses comparable in type and strength were neither seen in the cochlear nucleus nor in the superior olivary complex or in the medial geniculate nucleus.

Key differences between the stimulation-dependent responses of hearing and deaf brains were quantified. By counting neurons in the entire CIC cross-sections containing Fos protein contralateral to implantation with or without EIS, a significant but transient rise of their number was observed after 1 d of EIS of hearing and deaf rats (**Figure 4M**: all groups n.s. against each other except against group “1 d EIS nh,” which was significantly different to all other hearing groups with *p* < 0.001 and *p* = 0.027 for 1 d EIS vs. 7 d EIS, and group “1 d EIS deaf,” which was significantly different compared to all others with *p* < 0.001). Despite ongoing stimulation, Fos expression was no longer above control level by 7 d of EIS of hearing or deaf rats.

The density of GFAP staining was quantified on both sides of the colliculus in all experimental groups, calculating the contralateral-to-ipsilateral ratio of staining intensity. The intensity changed significantly toward the contralateral CIC in stimulated deaf brains after 1 and 7 d of EIS, whereas no asymmetry was detected after implantation only or in stimulated brains of hearing-experienced rats (**Figure 4N**: all groups n.s. against each other except against groups “EIS deaf,” with groups “1 d EIS deaf” and “7 d EIS deaf” significantly higher than all four nh groups and the two control groups of deaf rats with *p* < 0.001; as well as significantly different against each other with *p* = 0.0333). These observations clearly indicated that there is a quick and lasting modulation of the state of glial cells as a consequence of EIS in brains with a history of deafness, but are marginal in hearing-experienced brains at best.

### Glia Morphology Changes Due to EIS

**FIGURE 5:**
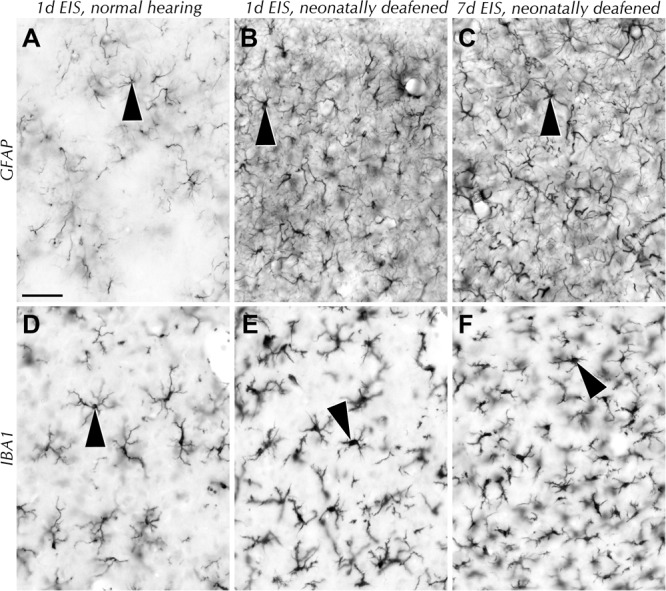
Glial cell populations in CIC after contralateral EIS. **(A)** In hearing-experienced rats, EIS did not induce changes in intensity and pattern of astrocyte density and arborization (arrowhead) compared to controls. **(B)** In deafened rats, 1 d EIS caused a dramatic increase in density of the dendritic network formed by hypertrophic astrocytes (arrowhead). **(C)** By 7 d EIS of deaf rats, hypertrophic astrocytes (arrowhead) still form a dense network with their processes. **(D)** In hearing-experienced rats, no changes were seen in the staining of microglial cells (arrowhead) after EIS of any duration. **(E)** In deaf rats, microglia (arrowhead) became stout and have retracted fine processes by 1 d EIS. **(F)** By 7 d EIS of deaf rats, microglia (arrowhead) have begun to extend fine processes again, increasing the density of the network. Scale bar: 50 μm.

The stimulation induced morphological transformations that occurred among different glial cell populations in the CIC of deafened rats deserved closer inspection. We noticed that both astrocytes and microglia changed their morphology in just those collicular regions where EIS induced strong Fos expression, but not elsewhere in CIC or in other regions of the midbrain. By 1 d of EIS, aggregation of astrocytes and their processes has greatly gained in prominence, mostly by the addition of processes (**Figure 5B**) compared to controls or stimulated hearing rats (**Figure 5A**). Six days later, the network is formed by thicker astrocytic dendrites arising from cell bodies that appeared to have been swollen compared to earlier stages (**Figure 5C**).

The reaction of microglia to EIS in deaf, but not or much less in hearing, brains followed the transition from a richly ramified to a more stout morphology (**Figures 5E,F**), i.e., from a resting to a reactive state (Walker et al., 2014). De-ramification of microglia is reported to be associated with their reaction to injury and inflammation, but in the case studied here de-ramification is incomplete as amoeboid microglia that might be indicative of phagocytic activity were rare at best. By 7 d of EIS, microglia were still arborized, apparently beginning to re-cover the space vacated by the initial retraction of their extensions by growth processes and possibly by mitosis (**Figures 5E,F**). This results in an obviously denser microglial network compared to the network observed after 1 d EIS of hearing-experienced or deafened rats (**Figures 5D–F**). Still, the question arose if the massive Fos expression and glia response to nerve stimulation in formerly hearing-inexperienced brains is related to cell death and, as a consequence, dedicated to debris management only.

Quantification of the IBA1 staining level above a gray tone threshold held constant throughout (**Supplementary Figure S2**) revealed significant changes against control levels for stimulated deaf rats only. It rose sharply within 1 d of EIS (*p* < 0.001) and declined until 7 d EIS (*p* < 0.001), still being above control level (*p* = 0.016) (**Figure 4O**). It might be noteworthy that there was a marginal rise of IBA1 staining after 1 d EIS in nh rats, although this change remained below the significance level.

### The Glia Response Is Not a Result of Cell Death

**FIGURE 6:**
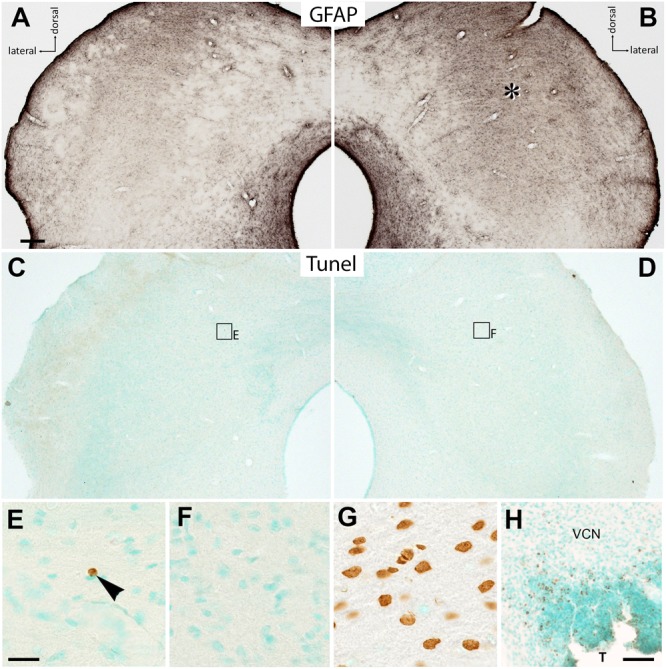
Absence of stimulation-related cell death. **(A,B)** CIC of a deafened rat by 1 d EIS and stained for GFAP, showing elevated staining levels contralateral to EIS (asterisk). **(C,D)** In a nearby section stained for Tunel, rare instances of positive staining unrelated in position to the region of high GFAP were found. **(E)** Frame in **(C)** at higher magnification showing a single Tunel-positive cell (brown nucleus) in CIC i to 1 d EIS (arrowhead). **(F)** In the region of high GFAP staining, Tunel-positive cells were equally rare. **(G)** Positive control of Tunel staining kit shows a lot of positive cells (brown). **(H)** Tunel staining in numerous cells (brown dots) bordering a tumor (T) that has grown in the ventral cochlear nucleus (VCN) in a rat not otherwise considered in this study. Scale bar for **(A–D)**: 200 μm; scale bar for **(E–G)**: 20 μm; scale bar for **(H)**: 100 μm.

One concern was that the central auditory system of a brain deprived from auditory stimulation was literally overwhelmed by a sudden, strong, and sustained activation of the sensory pathways entailing a massive release of glutamate not appropriately counterbalanced by inhibitory action so that excitotoxic effects may unfold. Indeed, not only microglia are involved in phagocytosis when cellular debris accumulates (Marín-Teva et al., 2004), but like neurons, glial cells are themselves vulnerable to glutamate insults (Matute et al., 2006). If this would be the context of microglia activation here observed, one had to expect stimulation-dependent cell death of neurons, glial cells, or both. To inquire into this possibility, we treated sections of brainstem tissue of hearing-experienced and deafened unstimulated and stimulated rats for the detection of DNA fragmentation indicative of apoptotic cell death. Particular attention was paid to the region of the CIC where Fos expression rose high and glia responses were massive (**Figure 4**), especially in contralateral CIC of stimulated deaf rats (**Figures 6A,B**). Tunel staining showed unequivocal instances of positive cellular nuclei in the brain sections, but these were extremely rare across the brainstem and failed to show a preferential localization in the auditory system (**Figures 6C–F**) or, more specifically, in the CIC contralateral to EIS (**Figure 6D**). Abundant staining for DNA fragments was obtained in positive controls (**Figure 6G**, brown nuclei) as well as in the immediate neighborhood of a tumor that has grown in the cochlear nucleus of a rat brain not otherwise included in the present study (**Figure 6H**, brown dots). This observation implies that neither neurons nor glial cells were affected by EIS in a way that would foster their decay even in hearing-inexperienced rats with an apparent massive activation upon auditory nerve stimulation. The morphological changes of glial cells that we identified in stimulated brains with a deaf history should therefore be related to tissue plasticity aiming to regain homeostasis and adjustment to novel levels and patterns of network activity, rather than with the disposal of cellular debris.

### Identity and Neighborhood of Fos-Positive Cells

**FIGURE 7:**
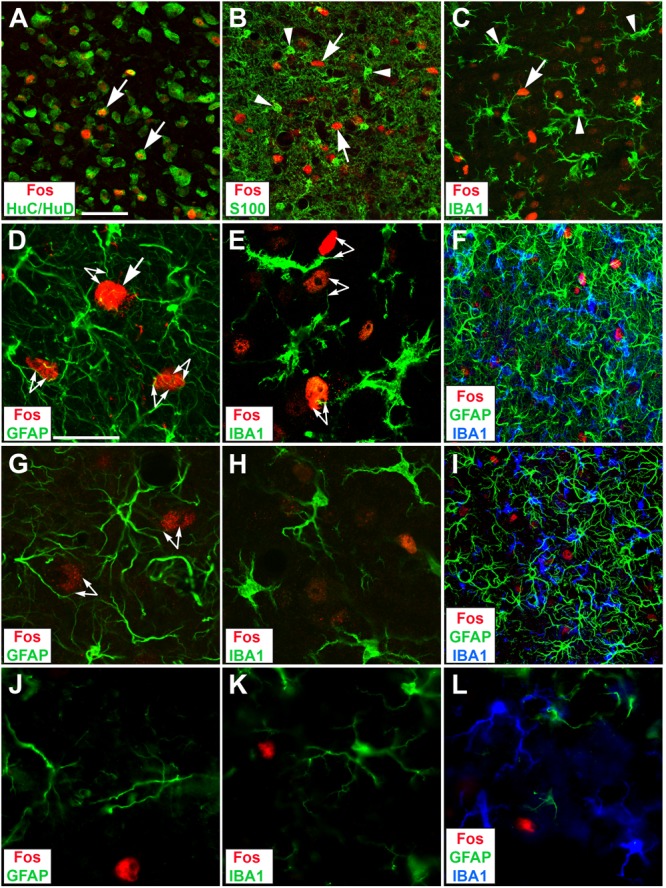
Identity and neighborhood of Fos-positive cells in the CIC of deafened rats by 1 **(B–F)** or 7 d EIS **(G–I)**, and of hearing rats by 1 d EIS **(J–L)** in confocal micrographs. **(A)** Whenever a collicular neuron contained a Fos-positive nucleus (arrows), it was located in HuC-/HuD-positive cytoplasm indicative of nerve cells. **(B)** Fos-positive nuclei (arrows) were never found in S100 positive cell bodies (some indicated by arrowheads) indicative of astrocytic cell bodies. **(C)** Fos-positive nuclei (arrow) were never found in IBA1 positive cell bodies indicative of microglia (some indicated by arrowheads). **(D)** Fos-positive cells (one indicated by arrow) were embedded in a meshwork of astrocytic processes that shared the neighborhood and showed distinct approximations (arrow-pairs). **(E)** Fos-positive cells were embedded in a dense population of microglia processes that showed specific spatial neuron–glia appositions (arrow-pairs). **(F)** The aggregations of glial processes were particularly dense where neurons showed strong Fos immunoreactivity and again showed many incidences of approximation between astrocytic processes and Fos-positive nuclei. **(G)** By 7 d EIS, Fos staining was waning and astrocyte aggregations have slightly eased. **(H)** By that time, microglia are still prominent but their acanthoid appearance seen by 1 d EIS **(E)** is no longer obvious. **(I)** At lower magnification a moderate decrease of glia aggregations can be recognized and approximations of glia processes and neurons recognizable by Fos staining are less evident by 7 d EIS compared to 1 d EIS **(F)**. **(J)** When nh rats were stimulated for 1 d astrocytic processes were sparse and approximations with Fos-positive neurons were rare. **(K)** Upon 1 d EIS, hearing rats did not change microglia morphology against controls, leaving them with thin long extensions that rarely touch Fos-positive cell bodies. **(L)** Sparse astrocytic extensions, thin microglial dendrites, and less frequent Fos-positive neurons provide few opportunities of approximations between them. Scale bar for **(A–C,F,I)**: 50 μm, scale bar for **(D,E,G,H,J,K,L)**: 20 μm.

Fos protein emerged exclusively in neurons. It was localized in the nuclei of cell bodies stained for HuC/HuD, a marker for neuronal cell bodies (**Figure 7A**, arrows and **Supplementary Video 1**). We failed to find cases of Fos protein staining in either astrocytes identified by S100 (**Figure 7B** and **Supplementary Video 3**) or microglial cells identified by IBA1 (**Figure 7C** and **Supplementary Video 2**) across all experimental groups.

Inquiring for the spatial relationship of Fos-positive cells to either type of glial cells in the contralateral CIC of deaf brains for 1 d, cases of close apposition of Fos-positive neurons and glial processes were found to be omnipresent (**Figures 7D,E**, arrow-pairs). This was true for microglia despite their reduced ramification, now touching neurons with rather stout processes (**Figures 7E**, arrow-pairs). Spatial approximations were obvious for astrocytes that have formed a dense meshwork (**Figures 7D,F**, arrow-pairs) and for the spiny microglia (**Figure 7E**) after 1 d EIS. These observations are consistent with trophic interactions among activated neurons and activated glial cells in deaf brains under EIS. After 7 d EIS, neuron–glia appositions are less obvious in deaf rats (**Figures 7G,H,I**, arrow-pairs). In hearing rats at 1 d EIS, appositions between Fos-positive cells and either type of glial cell are rare (**Figures 7J–L**).

### Modulation of Markers for Inhibitory and Excitatory Synapses

**FIGURE 8:**
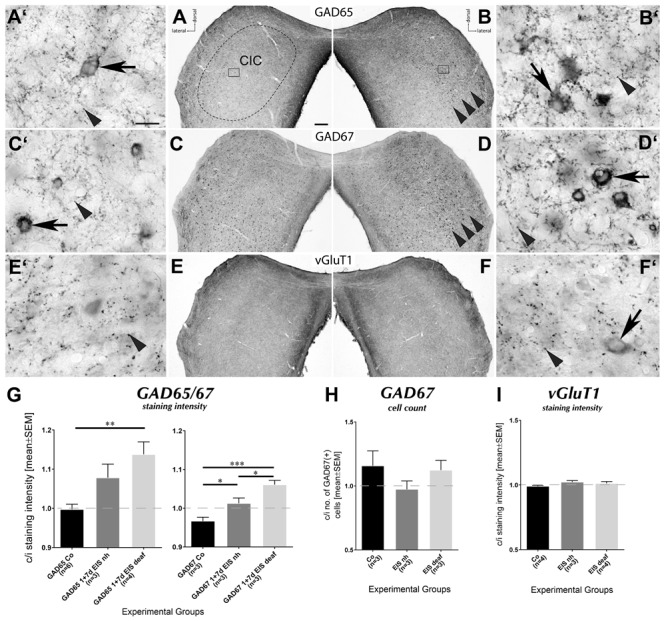
Modulation of GABAergic circuits by 7 d EIS in deafened rats. **(A,B)** Sections through CIC stained for GAD65. The network of stained fibers and boutons has increased contralateral to EIS (**B**, arrowheads) and the number of GAD65-positive cell bodies (**B′**, arrow), usually very rare in i CIC (**A′**, arrow), has increased. **(C,D)** Sections through CIC stained for GAD67. The network of stained fibers and boutons has increased contralaterally to EIS (**D**, arrowheads). Staining intensity of GAD67-positive cell bodies, normally present in considerable number (**C′**, arrow), has increased contralaterally (**D′**, arrow). **(E,F)** Sections through CIC stained for vGluT1. The network of stained fibers and boutons remained unchanged under EIS (**E′, F′**, arrowheads), with occasional vague indications of cell body staining (**F′**, arrow). Scale bar for **(A–F)**: 200 μm; scale bar for **(A′–F′)**: 50 μm. **(G)** Quantification of staining for GAD65 and GAD67 presented as contralateral-to-ipsilateral ratios. In the stimulated deaf rats, a significant rise of staining intensity contralateral to EIS was seen with GAD65 and GAD67 staining. For the stimulated hearing-experienced rats, significant differences were detected for GAD67 staining intensity compared to control group and the group of stimulated deaf rats. **(H)** Quantification of the number of GAD67-positive cell bodies. No significant changes occurred due to EIS. **(I)** Quantification of staining for vGluT1 presented as contralateral-to-i ratios that remained unchanged by unilateral EIS. nh, normal hearing; n, number of animals from which data were sampled.

Within days of bilateral deafening through cochlear ablation, neurotransmission changes in the CIC (Argence et al., 2006). In particular, GAD67 staining levels decreased therein within days of deafening and the inhibitory synaptic strength in the CIC declines (Vale and Sanes, 2002). This does not imply, however, that the molecular machinery supporting GABAergic transmission disappears. We found complex networks of fibers and a dense population of boutons by staining for GABA, GAD65, and GAD67 throughout hearing-experienced (not shown) and deaf brains including the CIC (cp. **Figures 8A′–D′**). Whereas GAD65 rarely showed stained cell bodies in hearing-experienced control or stimulated rats, the CIC of deafened rats developed a considerable number of such cell bodies under EIS (**Figure 8B**, arrowheads, **B′**, arrow). For GAD67 and GABA, positive cell bodies were seen in untreated or passively implanted controls of hearing and deaf rats at similar density and even after unilateral stimulation the number of GAD67 positive cell bodies in CIC revealed no changes in balance across the midline (**Figures 8C′,D′**, arrows, **H**: *p* > 0.5 for all comparisons).

Staining intensity of GAD65- and GAD67-positive boutons changed moderately from controls to hearing rats receiving EIS for 1 or for 7 d. By contrast, a pronounced unbalancing in GAD65-staining intensity occurred in the CIC contralateral to EIS in deaf rats (**Figure 8B** arrowheads, **B′,G** left plot: group “GAD65 1 + 7 d EIS deaf” is significantly darker stained than controls by *p* = 0.0024). Staining for GAD67 showed a similar trend (**Figure 8D** arrowheads, **D′,G** r plot: group “GAD67 1 + 7 d EIS deaf” is significantly stronger stained than controls by *p* < 0.001, and then group “GAD67 1 + 7 d EIS nh” by *p* = 0.0198; group “GAD67 1 + 7 d EIS nh” is significantly different from controls by *p* = 0.0235).

No stimulation-dependent changes were observed under any experimental treatment of hearing-experienced or deafened rats in pattern and density of vGluT1 staining (**Figures 8E–F′,I**: *p* > 0.5 for all comparisons).

### GABAergic Networks Involved in Network Remodeling

**FIGURE 9:**
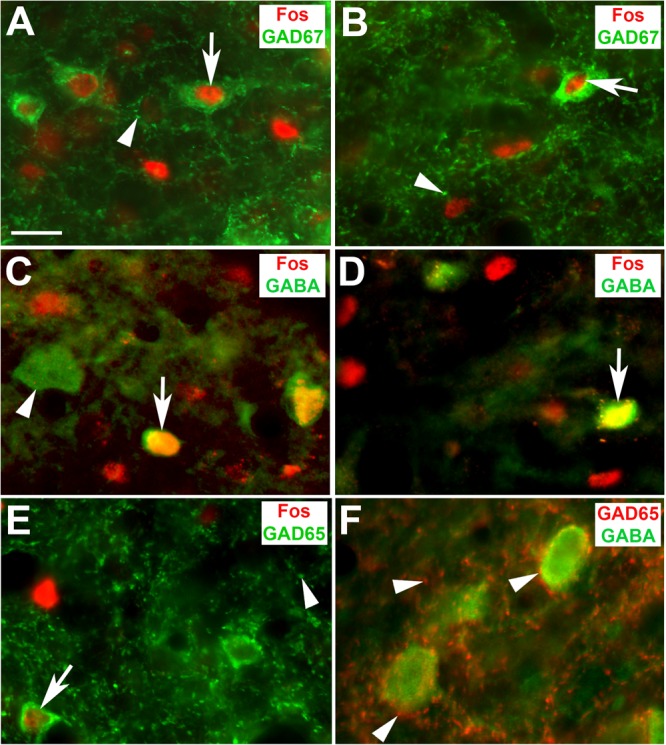
Fos in collicular GABAergic neurons of stimulated deaf rats. **(A,B)** Localization of Fos immunoreactivity (arrows) in cell bodies stained for GAD67 at 1 **(A)** and 7 d EIS **(B)**; arrowheads point to GAD67-positive boutons thought to be presynaptic endings. **(C,D)** Fos immunoreactivity in cell bodies stained for GABA (arrow) at 1 **(C)** and 7 d EIS **(D)**. Arrowhead points to Fos-negative GABA-stained cell body. **(E)** Fos immunoreactivity in a neuronal cell body stained for GAD65 (arrow) that emerged after 1 d EIS of deaf rats. **(F)** Inhibitory innervation (GAD65) of GABAergic neurons at 1 d EIS. Arrowheads in **(E)** and **(F)** point to GAD65-positive boutons thought to be presynaptic endings. Scale bar: 20 μm.

In compliance with our previous study wherein we investigated cell-type specificity of the initial EIS-induced Fos expression (Reisch et al., 2007), we again failed to identify instances of Fos-positive nuclei in supposedly GABAergic cell bodies of hearing rats (not shown). Remarkably, this changed for rats lacking hearing experience (**Figures 9A–E**). In stimulated deaf brains, we found instances of Fos-GAD67 (**Figures 9A,B**, arrows), Fos-GABA (**Figures 9C,D**, arrows), and Fos-GAD65 (**Figure 9E**, arrow) co-localization, while Fos-positive nuclei outside cell bodies containing GAD or GABA still prevailed (**Supplementary Video 4**). As a result of chronic stimulation of deaf rats, GAD65-positive boutons in the contralateral CIC grew more prominent also on the cell bodies of inhibitory neurons (cp. **Figure 9F**, arrowheads).

## Discussion

**FIGURE 10:**
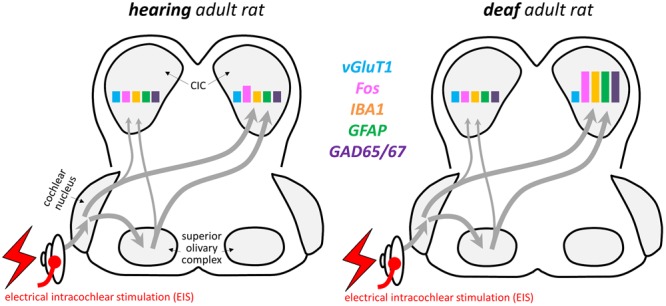
Summary diagram qualitatively comparing major molecular responses in the brains of hearing and deaf rats after EIS in CIC. Arrows show basic connectivity of the lower auditory brainstem.

The major finding of this study was the induction of a massive neuron–glia co-activation in the auditory midbrain of adult rats that were given first-time hearing experience by EIS after being neonatally deafened (**Figure 10**). We show that this response is unrelated to neuronal or glial apoptosis. For reasons of their local specificity and their temporal order with respect to the quick emergence of Fos in neurons and the slow increase of GAD65/67 staining, the observed glioplastic response is suggestive of forming a link of molecular signaling and cellular remodeling between neuronal spiking activity and the reactive local strengthening of inhibitory circuits that have remained immature.

### Neuronal Pathways

A major fraction of the ascending auditory pathway aiming for the CIC is glutamatergic (Kelly and Zhang, 2002; Fredrich et al., 2009). Neuronal excitation of collicular neurons upon acoustical (Ehret and Fischer, 1991) or electrical intracochlear (Illing et al., 2002; Reisch et al., 2007) stimulation is evident through their stimulation-dependent Fos expression. The axons transmitting sensory-evoked signals contact collicular neurons that carry AMPA and NMDA receptors (Kelly and Zhang, 2002) and belong to a neuronal population that consist to one-quarter of GABAergic cells (Merchán et al., 2005). Indeed, GABAergic neurons receive dense axosomatic inputs from terminals containing vGluT2 (Ito et al., 2009). These collicular neurons exert inhibitory effects both locally and by ascending projections (Peruzzi et al., 1997; Sivaramakrishnan et al., 2004; Li and Pollak, 2013).

Incoming neuronal excitation is balanced by local inhibition mostly carried by GABAergic cells as glycinergic cells are lacking from CIC (Merchán et al., 2005). When the brain is deprived of auditory activation for days or weeks, the molecular machinery serving GABAergic transmission is downregulated (Abbott et al., 1999; Argence et al., 2008) and the release of GABA upon electrical stimulation of the cochlear nerve proves to be markedly reduced compared to hearing animals (Bledsoe et al., 1995). However, when auditory nerve activity is re-invoked and maintained, the molecular machinery underlying neuronal inhibition recovers (Argence et al., 2008). However, processing of cochlear nerve activity is unlike that of hearing brains as the colliculi of neonatally deafened animals generate Fos-positive neurons in far greater number than nh litter mates (Nagase et al., 2000, 2003; Rosskothen-Kuhl and Illing, 2012).

### Involvement of Fos

The ascending auditory pathway is responsible for the stimulation-dependent Fos expression described here by releasing glutamate to be bound on diverse postsynaptic receptors. The most potent receptor agonist to induce postsynaptic Fos expression is AMPA, eliciting Fos expression in a dose-dependent manner (Soygüder, 2005). At a 1000-fold higher concentration NMDA also triggers Fos expression. With glutamate binding on NMDA receptors, but not on AMPA receptors, neurons release ATP (Khakh and North, 2012). ATP, in turn, triggers local outgrowth of microglia processes in mouse hippocampal brain slices (Dissing-Olesen et al., 2014).

Fos has been implicated in signaling cascades leading to apoptosis (Smeyne et al., 1993; Marcus et al., 1998). In adult rats deafened neonatally, we observed massive glia cell activation only in regions of extensive Fos expression upon electrical stimulation of their auditory pathway, immediately suggesting that extensive cell death is induced producing cellular debris that needed to be cleared by macrophages (Wu et al., 2008). However, when using Tunel staining, we proved that it failed entirely to indicate even marginal cell death. Obviously, the glia response in our experiments must be understood as serving an entirely different purpose.

While continuous stimulation losing its novelty resulted in the fading of Fos expression within days (**Figures 4B,C**), Fos mRNA expression persisted over 7 d EIS (**Figures 4E,F**). This was not the first incidence that transcription and translation of genes related to neuroplasticity can be seen to be only loosely linked. Following chronic EIS of rats, Gap43 mRNA is synthetized in CIC neurons without an obvious emergence of its protein (Rosskothen-Kuhl and Illing, 2014). Future research is needed to understand the reasons for a transcription of genes associated to neuroplasticity without their direct translation under EIS.

### Changes in Inhibition vs. Excitation after Deafness

Deafness entails an almost complete loss of spiking activity in ventral cochlear nucleus (Koerber et al., 1966), distributer of sensory-evoked activity into the central auditory pathways along different lines. After 30 d of deafness, the basal- and the stimulation-dependent release of GABA, but not of glycine, is reduced in the CIC (Bledsoe et al., 1995). After induced deafness in gerbils, the inhibitory synaptic strength in the CIC declines (Vale and Sanes, 2002) and GAD67 staining levels decrease within days (Argence et al., 2006). Simultaneously, EPSP amplitudes increase while excitatory transmitter release decreases among CIC neurons (Vale and Sanes, 2002). Lasting silencing of spiking activity reduces the number of inhibitory synapses and functional neural inhibition in organotypic cerebellar cell culture (Seil and Drake-Baumann, 1994). When spiking activity is reactivated, the affected neuronal network develops a transient hyperactivity. However, the neuronal network in the CIC of rats deafened at adulthood recovers lowered GAD67 levels as well as the glycine receptor GlyRα1 to normal within days of sustained electrical activation of the cochlear nerve (Argence et al., 2008). Our observations on the expression levels of GAD65 and GAD67 in neonatally deafened rats stimulated for 1 or 7 d are in consent with these reports but additionally demonstrate for the first time that this readjustment is even possible in an adult animal with a long history of deafness. We show that the modulation of GABAergic systems is not the result of a changing number of GAD67 positive neurons but rather of readjustments of the GABA-related metabolism within existing neurons and increasing GAD expression, possibly also causing retraction or sprouting of their dendrites and loss or formation of GABAergic synaptic contacts.

These data indicate that there is a connection between sensory-evoked neuronal spiking activity and the strength of inhibition within the inexperienced auditory network. However, it is not yet understood what might form the causal bridge between them.

### The Involvement of Astrocytes

Astrocytes are known to play a critical role in functions of neuronal networks (Liu et al., 2004). We observed astrocytes to grow in collicular regions where neuronal Fos expression is high. Their growth appears to be initiated by their exposition to high levels of glutamate released from ascending sensory afferents. Glutamate uptake by astrocytes goes through specific transporter proteins (GLAST and GLT-1) (Fonseca et al., 2005). If this uptake is not rapid enough, neurons die due to excitotoxicity.

Moreover, astrocytes are affected by extracellular glutamate through mGlu3 receptors. The intracellular IP3 signaling cascade thus triggered leads to astrocytic release of ATP into extracellular space (Khakh and North, 2012). Besides neurons, astrocytes are a second source that may contribute to elevated levels of ATP in extracellular space (Newman, 2003), specifically in regions where extracellular glutamate levels are high and apt to open neuronal NMDA receptors. This ATP availability may now affect local microglia known to carry purine receptors.

### The Involvement of Microglia

ATP has been identified as a chemoattractant for microglia (Davalos et al., 2005). Rapid growth of fine microglia extensions toward the source of ATP is mediated by their P2Y G-protein-coupled ATP receptors (Davalos et al., 2005). In the visual system, P2Y12 receptors of ramified microglia play an important role for activity-dependent plasticity of neurons in the adolescent brain (Sipe et al., 2016) and their interaction is modulated by visual experience (Tremblay et al., 2010). Moreover, ATP binding to the purinergic receptor P2X4R is supposed to be a major stimulus for BDNF to be synthetized and released from microglia (Trang et al., 2009; Parkhurst et al., 2013). We found that microglia increase expressing P2X4R upon their lesion-induced activation in the auditory brainstem (Illing et al., unpublished data). Microglial BDNF, in turn, increases phosphorylation of neuronal TrkB, a key mediator of synaptic plasticity.

### The Involvement of BDNF and GABAergic Transmission

With rise of BDNF in the extracellular space, the local GABAergic transmission changes. BDNF increases the amount of presynaptic GAD65 in the murine superior colliculus and enhances GABAergic transmission at high firing frequency (Henneberger et al., 2005). Similarly, BDNF promotes dendritic and synaptic development of GABAergic neurons in visual cortex (Palizvan et al., 2004). The effect of BDNF on generation and effectiveness of GABAergic transmission is further influenced by local astrocytes, stimulating BDNF binding to TrkB receptors (Elmariah et al., 2005) and taking up GABA from the extracellular space (Goubard et al., 2011). The activity-dependent emergence of GAD65-positive cell bodies in the contralateral CIC here reported (**Figure 8B**′, arrow) suggests that the demand of this GAD65 isoform became so strong that the expression of its gene *GAD2* in the cell body temporarily surpasses capacities of the cell’s axonal transport.

Based on our observations and recent literature, we propose that it is the joined glia cell activation and their specific activities that translate neuronal excitation into locally balanced neuronal inhibition.

## Conclusion

When neurons fire strong enough to induce Fos expression, they may trigger local responses of glial cells serving network homeostasis. By their local specificity and their temporal order, the stimulation-dependent response of microglia and astrocytes appears to constitute a bridge from neuronal firing activity to the local strengthening of synaptic inhibition. We suppose that our results may not only be applicable to cases of sensory dysfunction, but glial cell responses in the neighborhood of a limited number of neurons expressing Fos in nh rats may not have been reportable because the signals unfolding there are too subtle to be detected by methods we here applied. It remains to be seen if a glia link also serves to keep excitatory/inhibitory balances beyond the midbrain. As astrocytes and microglia can be specifically targeted by pharmacological intervention (Matute et al., 2006; Graeber and Streit, 2010; Toulme et al., 2010), therapeutical means may be designed to adjust or re-adjust excitatory/inhibitory balances wherever they are troubled (Nelson and Valakh, 2015).

## Author Contributions

NR-K and R-BI designed the study. NR-K and HH did the experiments. NR-K, HH, and R-BI evaluated the data. RB produced the RNA probes and verified their specificity. R-BI wrote the article. All authors approved the final manuscript.

## Conflict of Interest Statement

The authors declare that the research was conducted in the absence of any commercial or financial relationships that could be construed as a potential conflict of interest.

## Supplementary Material

FIGURE S1 Fos expression in the contralateral CIC of deafened rats after 7 days EIS. Neurons with Fos protein positive nuclei (**A**, Fos immunohistochemistry = IHC, dark dots) or *fos* mRNA positive neurons (**B**, Fos in situ hybridization = ISH, purple staining) of different staining intensity are spread over the dorsolateral part of the CIC (arrowheads). Scale bar: 200 μm.

FIGURE S2 Microglia quantification in the contralateral CIC. Representative examples of automatically detected IBA1 staining (black area) in a 40× photograph of a deafened control rat **(A)** and a deafened rat after 1d EIS **(B)**. Scale bar 50 μm.

FIGURE S3 fos *in situ* quantification compared to Fos immunohistochemistry quantification from sections through CIC contralateral to EIS after 1 and 7 days.

VIDEO 1 3D movie taken from *z*-stack of double immunofluorescent sections with Fos-positive nuclei (red) and HuC/HuD-positive neurons (green) in the contralateral CIC of a deafened rat under 1 day EIS. At the end of the movie, the red channel is switched off and on to uncover HuC/HuD staining behinds Fos positive nuclei.

VIDEO 2 3D movie taken from *z*-stack of double immunofluorescent sections with Fos-positive nuclei (red) and IBA1-positive microglia (green) in the contralateral CIC of a deafened rat under 1 day EIS. At the end of the movie, the red channel is switched off and on for an undisturbed view of the IBA1 staining.

VIDEO 3 3D movie taken from *z*-stack of double immunofluorescent sections with Fos-positive nuclei (red) and S100-positive astrocytes (green) in the contralateral CIC of a deafened rat under 1 day EIS. At the end of the movie, the red channel is switched off and on for better view of the S100 staining.

VIDEO 4 3D movie taken from *z*-stack of double immunofluorescent sections with Fos-positive nuclei (red) and GAD67-positive neurons (green) in the contralateral CIC of a deafened rat after 1 day EIS. At the end of the movie, the red channel is switched off and on for better identification of double staining.
