# Supplementary figures and images for: Astrocyte Hypertrophy and Microglia Activation in the Rat Auditory Midbrain Is Induced by Electrical Intracochlear Stimulation
Source: Front Cell Neurosci. 2018 Feb 22;12:43. doi: 10.3389/fncel.2018.00043 (PMC5827675; doi:10.3389/fncel.2018.00043)

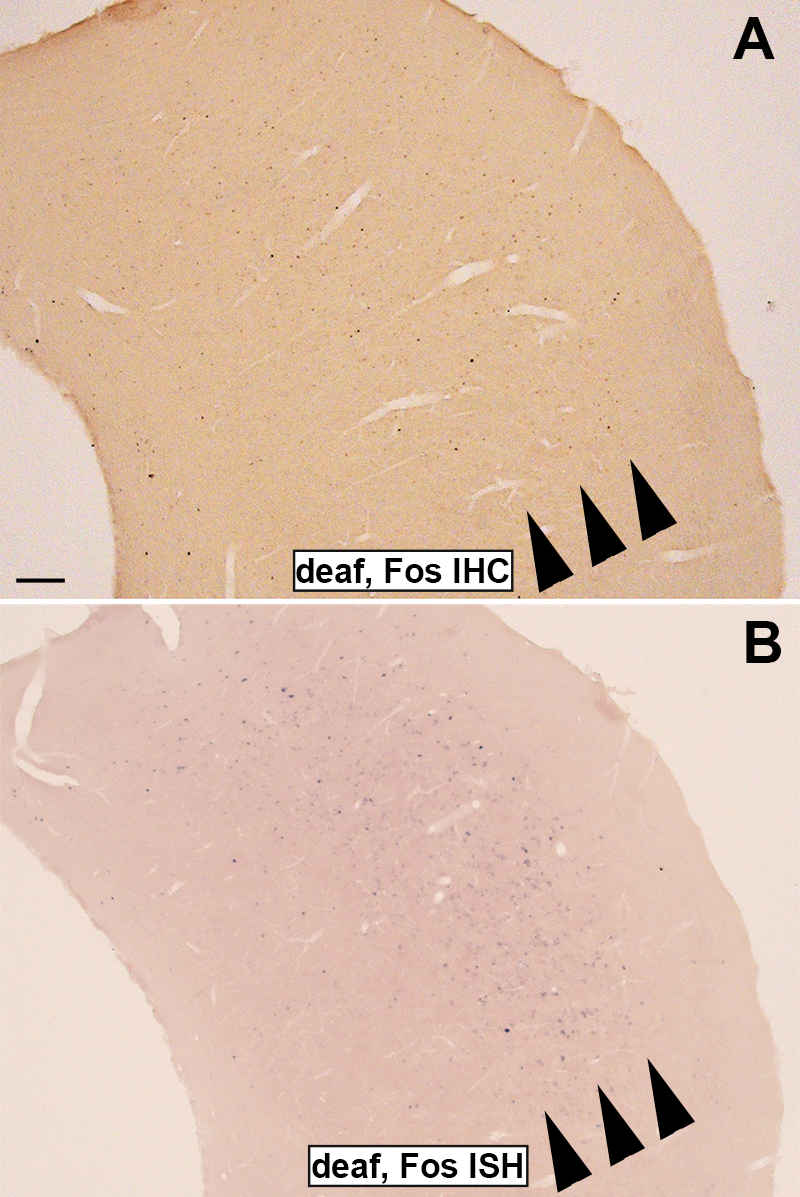

Supplement: FIGURE S1 — Fos expression in the contralateral CIC of deafened rats after 7 days EIS. Neurons with Fos protein positive nuclei (A, Fos immunohistochemistry = IHC, dark dots) or fos mRNA positive neurons (B, Fos in situ hybridization = ISH, purple staining) of different staining intensity are spread over the dorsolateral part of the CIC (arrowheads). Scale bar: 200 μm. [file Image_1.tif]

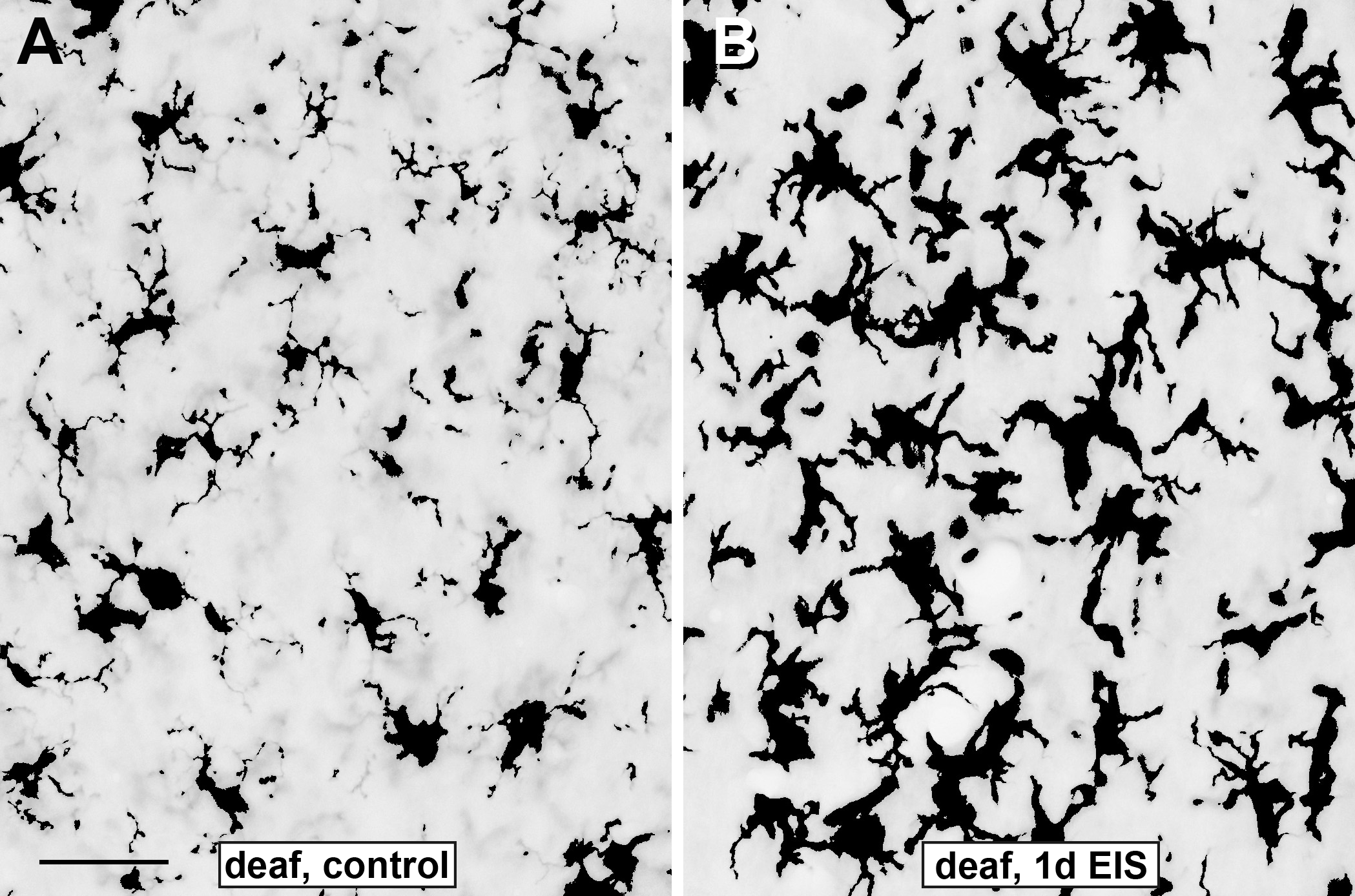

Supplement: FIGURE S2 — Microglia quantification in the contralateral CIC. Representative examples of automatically detected IBA1 staining (black area) in a 40× photograph of a deafened control rat (A) and a deafened rat after 1d EIS (B). Scale bar 50 μm. [file Image_2.tif]
